# Structural and Functional Analysis of the N-terminal Domain of the *Streptococcus gordonii* Adhesin Sgo0707

**DOI:** 10.1371/journal.pone.0063768

**Published:** 2013-05-17

**Authors:** Åsa Nylander, Gunnel Svensäter, Dilani B. Senadheera, Dennis G. Cvitkovitch, Julia R. Davies, Karina Persson

**Affiliations:** 1 Department of Odontology, Division of Oral Microbiology, Umeå University, Umeå, Sweden; 2 Department of Oral Biology, Faculty of Odontology, Malmö University, Malmö, Sweden; 3 Dental Research Institute, University of Toronto, Toronto, Canada; 4 Department of Chemistry, Umeå University, Umeå, Sweden; University of Kansas Medical Center, United States of America

## Abstract

The commensal *Streptococcus gordonii* expresses numerous surface adhesins with which it interacts with other microorganisms, host cells and salivary proteins to initiate dental plaque formation. However, this Gram-positive bacterium can also spread to non-oral sites such as the heart valves and cause infective endocarditis. One of its surface adhesins, Sgo0707, is a large protein composed of a non-repetitive N-terminal region followed by several C-terminal repeat domains and a cell wall sorting motif. Here we present the crystal structure of the Sgo0707 N-terminal domains, refined to 2.1 Å resolution. The model consists of two domains, N1 and N2. The largest domain, N1, comprises a putative binding cleft with a single cysteine located in its centre and exhibits an unexpected structural similarity to the variable domains of the streptococcal Antigen I/II adhesins. The N2-domain has an IgG-like fold commonly found among Gram-positive surface adhesins. Binding studies performed on *S. gordonii* wild-type and a Sgo0707 deficient mutant show that the Sgo0707 adhesin is involved in binding to type-1 collagen and to oral keratinocytes.

## Introduction

Oral streptococci are the most abundant bacteria in the oral cavity and approximately 70% of early colonizers belong to the streptococcal family [1]. Colonization occurs through bacterial adherence to molecules in the saliva-derived pellicle which covers all surfaces in the oral cavity. The salivary pellicle contains proteins, peptides and other molecules and so far 130 proteins have been identified [2]. The bacteria utilize a variety of cell surface proteins in order to adhere to a surface and thereby avoid clearance through swallowing. After adhesion, the colonizing bacteria themselves present new surfaces for adhesion by secondary colonizers. The commensal strain *Streptococcus gordonii* expresses an array of surface adhesins, for example the Antigen I/II (AgI/II) proteins SspA and SspB, and CshA and CshB that mediate interactions with salivary agglutinin (gp340) [3], [4]. Other examples are the two serine-rich cell surface glycoproteins GspB and Hsa that have a large number of binding partners, such as salivary MUC7 [5], secretory IgA [6], gp340 [7] and the platelet glycoprotein Ibα [8]. Although *S. gordonii* is mainly beneficial for oral health, the bacteria can become pathogenic if they spread to non-oral sites such as the heart valves with infective endocarditis as the result [9].

Surface adhesins on Gram-positive bacteria can adopt very different structures and depend on different forms of bioassembly. Many bacteria express pili which are long polymers of covalently linked pilins with an adhesin presented at the tip [10]. In contrast, the AgI/II adhesins expressed by oral streptococci adopt a unique monomeric structure where a central variable domain is presented as the tip on a stalk formed by intertwining flanking regions [11]. A third form of surface adhesins is built up from an N-terminal adhesion-mediating domain presented on a fibrillar stalk formed by a number of C-terminal repeat units as described for *e.g.* the *S. gordonii* adhesins CshA and GspB [12], [13], the *Streptococcus parasanguinis* adhesin Fap1 [14] and the *Streptococcus pyogenes* adhesin Epf [15]. A common feature among all these proteins is the presence of a C-terminal LPXTG-like sorting motif which is recognized by the enzyme sortase A (SrtA). This enzyme covalently links the protein to the cell wall. In a study on a *S. gordonii* SrtA deletion mutant, four previously unidentified LPXTG-containing surface proteins were detected at high levels by the examination of excreted proteins [16].

**Figure 1:**
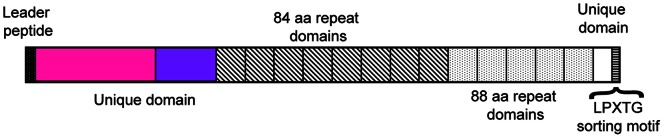
Domain architecture of Sgo0707. Schematic drawing of the domain architecture of Sgo0707. The 1643-amino acid long protein consists of several regions: a leader peptide, a unique N-terminal domain, a number of repeat sequences, a short unique C-terminal domain and an LPXTG sorting motif.

One of the proteins identified, Sgo0707, was present in high amounts and was predicted to be a fibrillar adhesin. The protein precursor for Sgo0707 is 1643 amino acids long and can be divided into several regions by examination of the sequence. A leader peptide is followed by an N-terminal domain of 419 amino acids. Next follows an 84-amino acid sequence that is repeated eight times followed by an 88-amino acid sequence, repeated five times. Prior to the LPXTG sorting motif and transmembrane helix is a small unique domain (Fig. 1). We hypothesize that the N-terminal domain functions as the adhesin and that the repeat domains build up the stalk of the protein, similar to that described previously for streptococcal adhesins [12], [13], [14], [15].

To add to our knowledge regarding the structure and function of adhesins from Gram-positive bacteria in general and oral streptococci in particular, we have solved the crystal structure of the N-terminal domain from *S. gordonii* Sgo0707 to 2.1 Å resolution. We also present the result of binding studies of Sgo0707 to keratinocytes, serum, saliva, type-1 collagen and a set of glycan structures.

## Materials and Methods

### Bacterial Strains and Culture Conditions

Wild-type *S. gordonii* DL-1 (Challis), and the ΔSgo0707 strain, stored at –70°C in skim milk (Oxoid), were grown on blood agar in 5% CO_2_ in air at 37°C for 24 h. For biofilm experiments, inocula were prepared by transferring colonies into modified *Actinomyces* defined medium (m-ADM) and incubating in an atmosphere of 5% CO_2_ in air at 37°C overnight. Erythromycin was added to the medium at a concentration of 50 µg/ml for ΔSgo0707. Aliquots (500 µl) were transferred to 4.5 ml fresh m-ADM and incubated in 5% CO_2_ in air at 37°C; the OD_600_ was monitored until the mid-exponential growth phase (OD_600_ = 0.6±0.1) was reached. Cells were harvested by centrifugation (3000 *g*, 5 min at 4°C), washed in 10 mM PBS (pH 7.5) and re-suspended in m-ADM to a cell concentration of 1×10^7^/ml.

### Cloning of the Sgo0707 N-terminal Domain

Residues encoding 36–458 from *sgo0707* (UniProt A8AW49) were amplified by PCR using forward primer 5′-aaaaa*ccatgg*cgttagaagagattaaaaat-3′ and reverse primer 5′-ttttt*ggtacc*ttacttttcataaagaagagc-3′. The PCR product was digested with *NcoI* and *Acc65I* (restriction sites in italics) and ligated into the corresponding sites of a pET-M11 expression vector containing a His-tag at the N-terminus. The final construct, Sgo0707N, encodes MKHHHHHHPMSDYDIPTTENLYFQGAM-Sgo0707_36–458_. The plasmid was transformed into *Escherichia coli* DH5α and subsequently selected on kanamycin plates. The positive clones were verified by DNA sequencing.

### Overexpression and Purification of Sgo0707N

The Sgo0707N construct was overexpressed in *E. coli* BL21 and grown in Luria Broth supplemented with 50 µg/ml kanamycin. The culture was grown at 37°C until it reached an OD_600_ of 0.6 when the temperature was lowered to 28°C and protein expression was induced with 0.5 mM IPTG. The culture was allowed to grow for an additional 4 h. Cells were harvested by centrifugation at 5300 *g* for 20 min and the pellets were stored at -80°C. Pellets were resuspended in lysis buffer (20 mM Tris pH 7.5, 150 mM NaCl and 10 mM imidazole pH 8.0) supplemented with EDTA-free protease inhibitor cocktail (Roche). The cells were lysed on ice by sonication and cellular debris was removed by centrifugation at 39000 *g* for 35 min. The supernatant was loaded onto a Ni-NTA agarose column (Qiagen). The column was washed with lysis buffer containing 20 mM imidazole and eluted in the same buffer containing 300 mM imidazole. The protein was then concentrated and the buffer exchanged to 20 mM Tris pH 7.5 and 0.5 mM EDTA. The protein was further purified on an UNO Q6 anion exchange column (Bio-Rad) equilibrated with 20 mM Tris pH 7.5 and eluted with a gradient of the same buffer containing 1 M NaCl. Finally the protein was purified using size exclusion chromatography on a Superdex200 16/60 PG column (Amersham Biosciences) equilibrated with 20 mM Tris pH 7.5, 200 mM NaCl and 1 mM EDTA. The protein was concentrated to 84 mg/ml in 20 mM Tris pH 7.5.

Selenomethionine (SeMet)-substituted Sgo0707N was expressed in *E. coli* BL21 grown in M9 medium supplemented with glucose at 37°C. At an optical density of ∼0.6 at 600 nm, lysine, threonine, phenylalanine at 100 mg/l, leucine, isoleucine, valine, proline and SeMet at 50 mg/l were added to down-regulate the methionine synthesis [17]. The temperature was lowered to 20°C and the expression was induced with 0.5 mM IPTG. The culture was grown overnight. The SeMet labelled protein was purified as described above with the exception that 0.5 mM Tris (2-carboxyethyl)-phosphine hydrocholoride (TCEP) was present throughout all steps. The SeMet protein was concentrated to 92 mg/ml.

### Construction of the *sgo0707* Deletion Mutant

A knockout mutant of *sgo0707* (strain ΔSgo0707) was constructed using a PCR-ligation mutagenesis strategy, as previously described [18]. Briefly, genomic DNA from *S. gordonii* DL-1was used to amplify the upstream flanking region of *sgo0707* using P1*:* 5′-tcagccataccaccgtcaac-3′ and P2: 5′-*ggcgcgcc*acaaagccgaagccaaacc-3′ primers, whereas the downstream flanking region was amplified using primers P3: 5′-*ggccggcc*accatcagcccactcaatg-3′ and P4: 5′-tgctggtaaagacggttgg-3′ (italicized bases represent *AscI* and *FseI* cut sites, respectively). After digesting with respective restriction enzymes, both amplicons were ligated with an erythromycin resistant cassette with exposed *AscI* and *FseI* cut sites. The ligated product was amplified using P1 and P4 primers, transformed into wild-type cultures, and selected for resistance to erythromycin. Successful mutagenesis was validated using PCR and nucleotide sequence analyses.

### Preparation of Cell Wall Proteins and 2DE

Mid-exponential growth phase cells were washed with PBS and centrifuged at 2000 *g* for 10 min at 4°C. The pellet was then re-suspended in 0.2% sulfobetaine (3–10) and shaken for 1 h at 100 rpm, 28°C before being centrifuged at 6000 *g* for 10 min at 4°C. After washing three times in ultrapure water, the cells were re-suspended in spheroplasting buffer (20 mM Tris-HCl, pH 6.8, 10 mM MgCl_2_, 26% w/v raffinose), 100 U/ml mutanolysin (Sigma) added, and the sample incubated for 75 min at 37°C by 20 min at 60°C. Samples were placed on ice before being centrifuged at 12000 *g* for 20 min at 4°C. The supernatant was then dialysed against ultrapure water and freeze-dried. The resulting material was dissolved in two-dimensional polyacrylamide gel electrophoresis (2DE) rehydration buffer (8 M urea, 2% CHAPS, 10 mM DTT, 2% immobilized pharmalyte gradient buffer (GE Healthcare Life Sciences)) and stored at −20°C until subjected to 2DE and identification with liquid chromatography-tandem mass spectroscopy (LC-MS/MS) as described previously [16].

### Adherence of *S. gordonii* DL1 Wild-type or ΔSgo0707 Strain to Protein-coated Surfaces

Binding of the wild-type or mutant strain to different surface coatings was investigated in an ibiTreat µ-slide VI flow-cell model. Stimulated whole saliva, pooled from six individuals was prepared according to the method described by Palmer and co-workers [19]. 5% human serum was prepared by diluting human serum (Lonza Group Ltd) with distilled water. Human type-1 collagen (Coating matrix kit, GIBCO) was used according to the manufacturer’s instructions. Flow-cells were coated overnight at room temperature with 100 µl of each protein preparation (saliva, serum, or collagen) to create conditioning films and gently rinsed with 3×100 µl m-ADM before use. Mid-exponential growth phase cells of the wild-type strain and the ΔSgo0707 strain were passed over the flow-cell surfaces for 2 h at a rate of 3.6 ml/h. The flow-cells were subsequently rinsed with m-ADM at the same flow rate for 30 min to remove non-attached cells. The LIVE/DEAD® *Bac*light™ Bacterial Viability Kit was used to stain the adhered cells which were then visualized with confocal laser scanning microscopy (CLSM) (Nikon Eclipse TE2000-E confocal microscope). Twenty images were collected in each experiment and experiments were repeated three times using independent bacterial cultures. Image analysis was performed using the *bio*Image_L software package [20]. The mean surface coverage for the wild-type and ΔSgo0707 strains was compared using student’s t-test.

To investigate the inhibitory effect of exogenous Sgo0707N recombinant protein on binding of wild-type *S. gordonii* DL1 to type-1 collagen coated surfaces, surfaces were incubated overnight at 37°C with recombinant Sgo0707N protein at three concentrations (0.01, 0.05 and 0.1 mg/ml). Wild-type cells of *S. gordonii* DL1 in M-ADM containing the same concentration of recombinant protein were then flowed over the surfaces and the adherence assessed as described above. The mean surface coverage in the presence of the three concentrations of the recombinant protein were compared with the control using ANOVA (n = 3).

### Adherence of *S. gordonii* DL1 Wild-type and ΔSgo0707 Strains to Oral Keratinocytes

Immortalized normal human keratinocytes OKF6/TERT-2 [21] were seeded into Ibidi µ-Slide VI ibiTreat flow-cell chambers (Ibidi GmbH, Germany) and allowed to grow until 30% confluence was reached as described previously [16].The keratinocyte layer was washed with 3×100 µl m-ADM before loading 100 µl of bacterial cultures in mid-exponential growth phase into each lane. After 1 h at 37°C in 5% CO_2_ in air, the lanes were washed with 3×100 µl PBS, pH 7.2 and stained with LIVE *Bac*Light™ Bacterial Gram Stain Kit (Invitrogen) which stains the Gram-positive bacteria red and the keratinocytes green. Adhered bacteria were visualized as above and twenty images were collected in each experiment. Experiments were repeated three times using independent bacterial cultures. Image analysis was performed manually by counting the number of keratinocytes and adhered bacteria in each image and calculating a ratio of bacteria/keratinocyte. For each of the three experiments, 1500–2000 keratinocytes were analysed for the wild-type or ΔSgo0707 strains respectively. The mean of adhered bacteria per thousand keratinocytes for the wild-type and ΔSgo0707 strains was compared using student’s t-test.

### Auto-aggregation Assay

The wild-type and mutant strains were grown to mid-exponential growth phase in Todd Hewitt broth and the degree of auto-aggregation assessed as described previously [22]. Briefly, cultures were centrifuged (3000 *g*, 5 min at 4°C), washed twice with PBS and then dispersed in co-aggregation buffer (1 mM Tris pH 8, 0.15 M NaCl, 0.1 mM CaCl_2_, 0.1 mM MgCl_2_ and 0.02% NaN_3_). The suspensions were vortexed for 10 s, adjusted to OD_660_ = 0.5 and 0.7 ml of bacterial suspension transferred to a sterile cuvette. The optical density was recorded. The cuvettes were allowed to stand at room temperature for 1 h before the optical density was recorded again. The degree of auto-aggregation was calculated as the percentage decrease in optical density over this time.

### Glycan Array

Recombinant Sgo0707N in 25 mM Tris pH 7.5 was concentrated and lyophilized (total amount 2.1 mg). The protein was dissolved in 20 mM Tris pH 7.4, 150 mM NaCl, 2 mM CaCl_2_, 2 mM MgCl_2_, 0.05% Tween 20 and 1% BSA to a concentration of 200 µg/ml. A total of 611 glycans in replicates of six were screened and binding was detected with an anti-his antibody. The array was performed by the Consortium for Functional Glycomics, Core H (https://www.functionalglycomics.org).

### Thermal Shift Assay

Sgo0707N was screened for stabilizing metal-buffer conditions using the thermofluor method [23]. In short, 25 µl solutions containing 3×Sypro Orange (Molecular Probes), 100 mM Tris pH 7.5, 6 mM metal and 7.5 mg/ml protein were added to 0.1 ml PCR tubes and heated in a qPCR detection system (Rotor-Gene 6000, Corbett Life Science) from 28°C to 95°C in increments of 0.2°C. Changes in fluorescence were monitored and the melting temperature (Tm) was determined by calculating the derivative of the midpoint of the protein unfolding transition. The metals that were screened were NaCl, LiCl, CaCl_2_, MnCl_2,_ MgCl_2,_ and Zn(OAc)_2_. The addition of water to an identical protein mix was used as a control. The experiment was performed in triplicate.

### Crystallization and Data Collection of Sgo0707N

Initial crystallization trials were performed by a Mosquito pipetting robot (TTP Labtech) using sitting-drop vapour diffusion and standard crystal screening kits (Hampton Research and Molecular Dimensions) in 96-well plate format. The initial hits were optimized using the sitting-drop method by mixing 2 µl of purified protein at a concentration of 15 mg/ml and 2 µl of reservoir solution. Crystals were initially obtained in condition A3 and G6 of the Structure Screen I/II (Molecular Dimensions). The final crystallization condition was optimized to 0.1 M sodium acetate pH 5.0, 0.2 M ammonium sulphate and 20% (w/v) polyethylene glycol 4000. Crystals grew within one week in space group *I*222 with cell dimensions a = 152.1, b = 158.1 and c = 164.3 Å. Crystals of the SeMet labelled protein were obtained from the same conditions. Prior to data collection the crystals were soaked in crystallization solution supplemented with 20% polyethylene glycol 400, mounted on loops, and vitrified in liquid nitrogen. Diffraction data were collected at 100 K. Data from both native and SeMet containing crystals were collected at beamline ID-23 at the European Synchrotron Radiation Facility, Grenoble, France to 2.1 and 2.4 Å resolution respectively. Data were processed with XDS [24] and scaled with SCALA from the CCP4 program suit [25].

### Structure Solution and Refinement

The SeMet-containing structure was solved with SAD-phasing using AutoRickshaw [26]. Density modification and automatic model building were performed using AutoRickshaw and ArpWarp [27] and resulted in a readily interpretable map. The model was further built using rounds of manual building in COOT [28] and refined using phenix.refine [29]. The native structure was solved by molecular replacement using the program Phaser [30], with the SeMet containing structure as the search model. The native structure was similarly refined with phenix.refine. The first refinement step included rigid body refinement and simulated annealing starting at 5000 K. Manual inspection, rebuilding and addition of water molecules were performed with COOT [31]. For refinement 5% of the reflections were removed for the calculation of R_free_. The quality of the model was analyzed with WHATCHECK [32] and Ramachandran Statistics were obtained from COOT. The model was subjected to four-fold NCS restraints throughout the refinement. Refinement statistics are given in Table 1. Figures were drawn with CCP4MG [33].

**Table 1:** Data collection, refinement and model quality statistics for Sgo0707.

	Native Sgo0707	SeMet Sgo0707
Data collection		
Space group	*I*222	*I*222
Cell dimensionsa, b, c (Å)	152.13, 158.12, 164.26	152.12, 157.40, 165.26
Wavelength (Å)	0.979300	0.979300
Resolution (Å)	48.45–2.09	48.47–2.39
Highest resolutionshell (Å)	2.20–2.09	2.52–2.39
Total reflections^a^	674,645 (57,281)	885,346 (76,170)
Unique reflections^a^	114,899 (15,141)	77,177 (10,121)
I/σ (I)^a^	13.9 (3.9)	22.8 (5.6)
R_sym_(%)a, b	8.0 (32.4)	8.6 (34.7)
Completeness (%)^a^	98.4 (89.8)	98.5 (89.8)
Overall redundancy	5.9 (3.8)	11.5 (7.5)
Refinement		
No. of reflections	112615	
Rwork/Rfree (%)^c^	17.9/22.5	
Average B-factors (Å^2^)		
Wilson plot	29.6	
Protein (Chain A/B/C/D)	27.6/28.6/30.0/31.6	
Water	35.8	
Metal ions	37.9	
Sulfate ions	48.5	
No. protein atoms	13424	
No. metal ions	4	
No. sulfate ions	8	
No. water molecules	1282	
RMSD from ideal		
Bond lengths (Å)	0.007	
Bond angles (°)	1.103	
Ramachandran plot		
Preferred, allowed,outliers (%)	93.88/5.36/0.77	

a Values in parentheses indicate statistics for the highest resolution shell.

b *R*_sym_ = Σ*_hkl_* Σ*_i_* |*I*_i_ *(hkl)* - <*I*(*hkl*)>|/Σ*_hkl_* Σ*_i_ I*_i_ (*hkl*), where *I_i_*(*hkl*) is the intensity of the *i*th observation of reflection *hkl and <I(hkl)>* is the average over of all observations of reflection *hkl.* ^c^ *R*_work_ = Σ | |*F*_obs_| - | *F*_calc_| |/Σ | *F*_obs_|, where *F*_obs_ and *F*_calc_ are the observed and calculated structure factor amplitudes, respectively. *R*_free_ is *R*_work_ calculated using 5% of the data, randomly omitted from refinement.

Structure factors and coordinates have been deposited in the Protein Data Bank (PDB; http://www.rcsb.org/pdb ) under accession code 4IGB.

### Docking of type-1 Collagen

Three web-based docking programs (GRAMMx [34], Firedock [35] and Hex Server [36]) were used to dock an 11 residue long type-1 collagen fragment (derived from PDB 2F6A [37]) to the Sgo0707 crystal structure.

## Results

### ΔSgo0707 Shows Reduced Binding to Type-1 Collagen but Not to Saliva or Serum-Coated Surfaces

**Figure 2:**
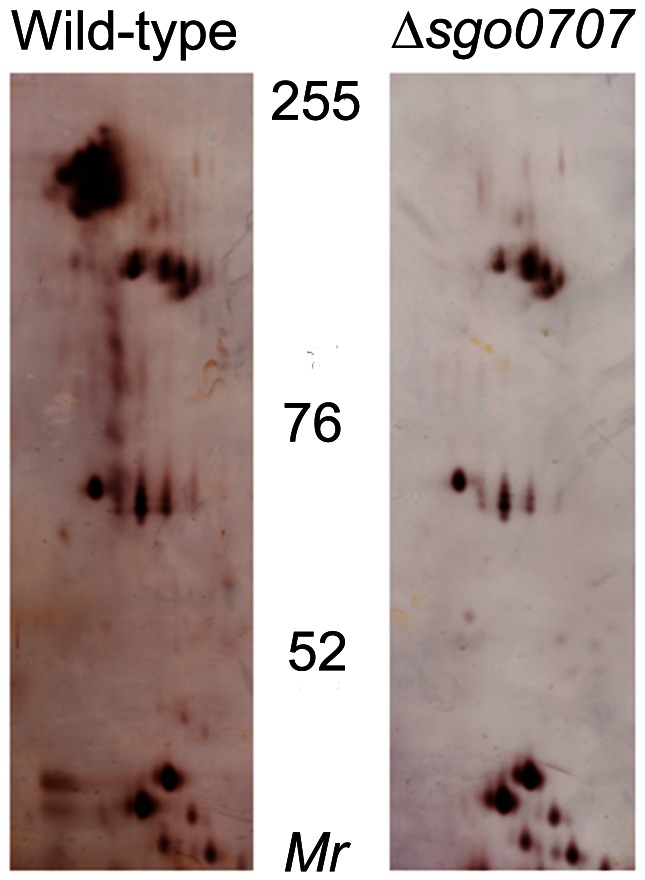
Silver-stained 2DE gels of cell wall proteins from wild-type and ΔSgo0707 strains of *S.gordonii*. Proteins prepared as described in materials and methods were subjected to isoelectric focussing at pH 4–7 followed by SDS-PAGE in 7% gels. The migration positions of molecular mass markers are shown.

To investigate the role of Sgo0707 as an adhesin, a mutant deficient in the protein was constructed in *S. gordonii* DL1 (ΔSgo0707). In order to confirm that Sgo0707, previously identified from the culture fluid of a *S. gordonii* DL1 sortase deficient mutant, was expressed in the cell wall of the wild-type strain, cell wall proteins were prepared and subjected to 2DE (Fig. 2). This revealed a prominent cluster of spots with M_r_ 130–180 kDa. Using mass spectroscopy, this cluster was identified as corresponding to Sgo0707. This cluster was absent from cell wall preparations from the ΔSgo0707 strain, thus confirming that the mutant did not express this protein.

**Figure 3:**
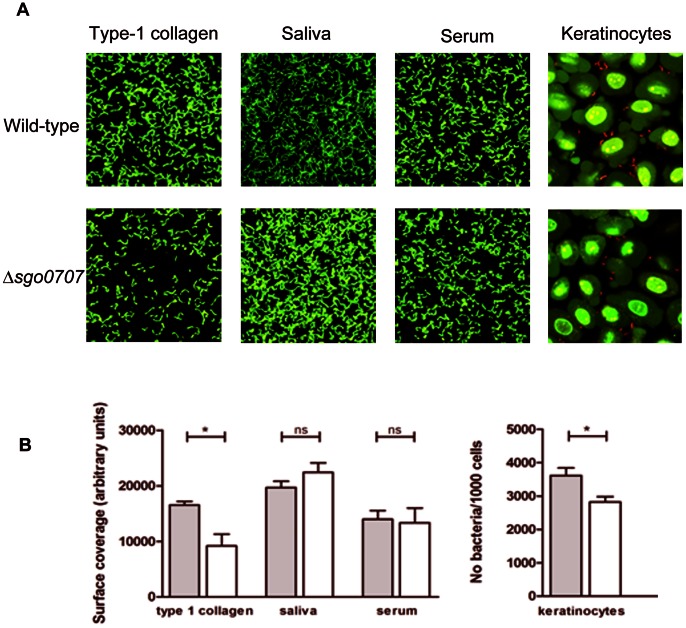
Role of Sgo0707 in *S. gordonii* binding to type-1 collagen, saliva, serum and oral keratinocytes. A: Fluorescence micrographs showing adherence of wild-type and ΔSgo0707 strains to protein-coated surfaces and to oral keratinocytes. In panels 1–3 bacteria were flowed over surfaces coated with type-1 collagen, 25% saliva or 10% serum for 2 h, as described, and adhered bacteria stained green using the LIVE/DEAD® *Bac*light™ Bacterial Viability stain. Images were obtained using CLSM and the surface coverage determined by image analysis using the *bio*Image_L software package. In panel 4, bacteria were flowed over keratinocyte layers for 1 h, stained using the LIVE *Bac*Light™ Bacterial Gram Stain Kit and viewed with CLSM. The mean number of bacteria (stained red)/1000 keratinocytes (stained green) was determined by manual image analysis. B: Graphs showing the adherence of the wild-type (grey bars) and ΔSgo0707 (white bars) strains to different surface coatings. For type-1 collagen, saliva and serum, adherence is expressed as surface coverage (arbitrary units) whereas for the keratinocytes, binding is expressed as number of bacteria/1000 keratinocytes. The mean and standard error of three independent experiments is shown (* indicates a significant difference p<0.05).

The wild-type and ΔSgo0707 strains were then compared for their binding to surface-associated proteins; type-1 collagen, saliva and serum (Fig. 3a). The wild-type strain bound well to all the coatings tested although adherence was slightly better to saliva than to type-1 collagen or serum. The mutant strain showed a 40% lower level of binding to collagen than its wild-type counterpart (p<0.05), whereas for saliva and serum, no such decrease was seen (Fig. 3b) This suggests that Sgo0707 is involved in binding of *S. gordonii* to type-1 collagen but not to saliva or serum-coated surfaces.

Wild-type *S. gordonii* also bound well to oral keratinocytes (mean number of bacteria per 1000 cells = 3602±439) while binding of ΔSgo0707 was 30% lower (mean number of bacteria per 100 cells = 2820±52). This difference was significant (p<0.01) (Fig. 3).

To determine whether Sgo0707 plays a role in cell-cell interactions in *S. gordonii*, the auto-aggregation of the wild-type and mutant strains was also investigated. The percentage auto-aggregation seen for the wild-type strain over 1 h was 9±0.6% while the corresponding value for the ΔSgo0707 strain was 11±0.9%. The difference between these values is not significant at the 5% level suggesting that Sgo0707 does not play a role in auto-aggregation.

### Binding of Sgo0707 to type-1 Collagen is Mediated via the N-terminal Domain

**Figure 4:**
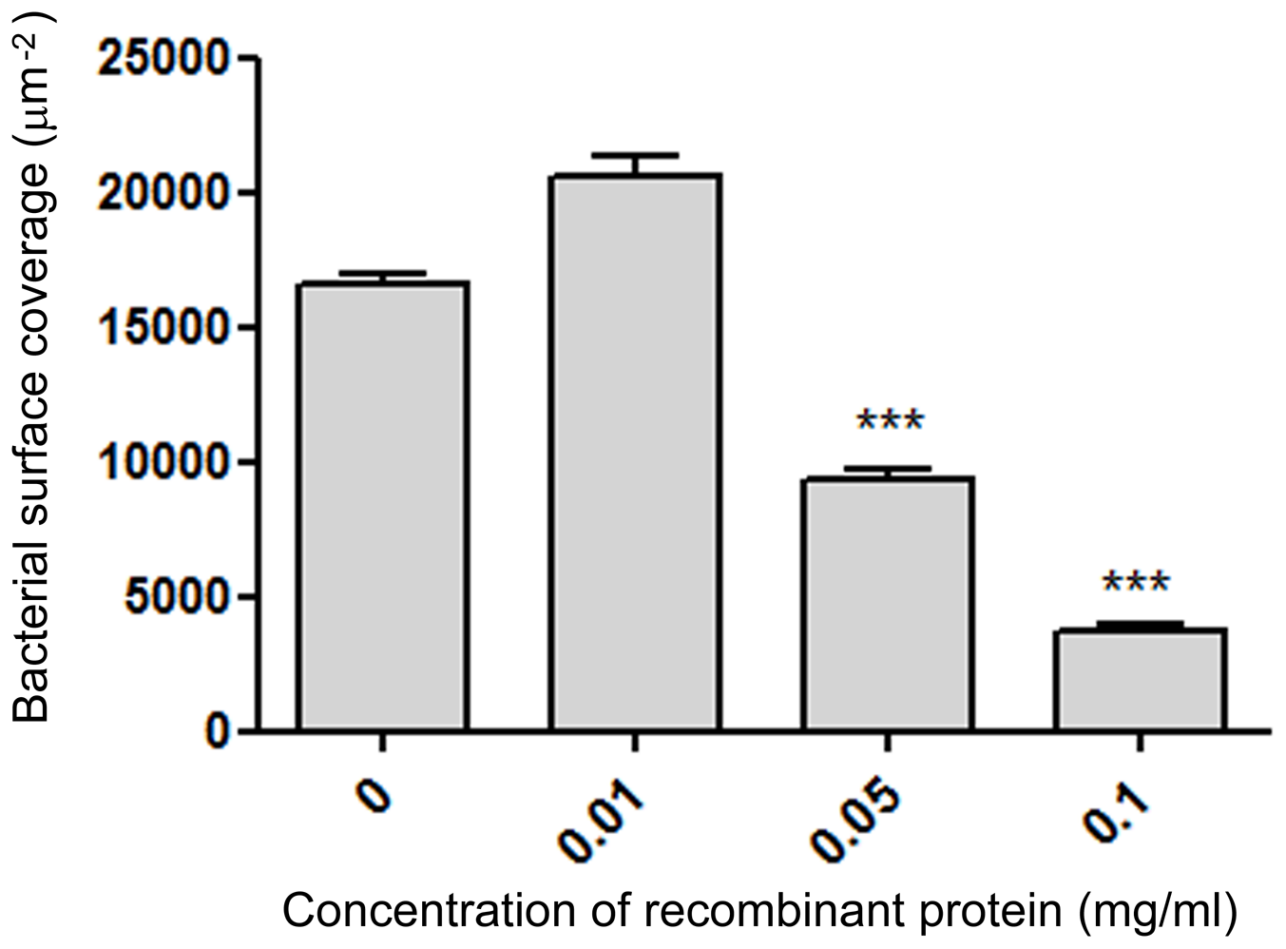
Inhibition of binding of *S. gordonii* DL1 to type-1 collagen coated surfaces using recombinant Sgo0707_36–455_ protein. Flow-cell surfaces were coated with type-1 collagen followed by different concentrations of recombinant Sgo0707_36–455_ protein. Bacteria were flowed over the surfaces for 2 h in the presence of the same concentrations of recombinant Sgo0707_36–455_ and adhered bacteria were visualized using the Baclight Live/Dead stain and analysed with *bio*Image_L software package. The mean and standard error of three independent experiments is shown (*** indicates a significant difference p<0.001 from control).

To test whether the Sgo0707 N-terminal domain mediates binding to type-1 collagen, recombinant Sgo0707N was overexpressed and the ability of the protein to inhibit binding was tested (Fig. 4). This showed that the protein competitively inhibited binding of wild-type *S. gordonii* in a concentration-dependent manner. However, inhibition of binding was not complete even at high concentrations of the recombinant protein.

### Carbohydrate Binding

In order to investigate whether or not Sgo0707N binds to carbohydrates, a glycan array screening was performed at the Consortium for Functional Glycomics, Core H. A total of 611 glycans (of mammalian and pathogen origin) were screened but no binding was detected for any of the sugars.

### Metal Stabilization

A thermally induced melting analysis was performed on Sgo0707N in combination with a set of mono- and divalent metals using the thermofluor method [23]. Results showed that the protein was most stable when supplemented with Ca^2+^ ions (Tm = 62°C). Other divalent ions (Zn^2+,^ Mg^2+^ and Mn^2+^) resulted in melting temperatures of 55–57°C whereas monovalent ions (Na^+^and Li^+^), gave the same melting temperature as the non-metal supplemented prot ein (Tm = 54°C). The melting temperatures are presented in Table 2.

**Table 2:** Protein melting temperatures when supplemented with metal ion solutions.

Additive (6 mM)	Tm (°C)
No additive (H_2_O)	54.4
Ca^2+^	62.4
Zn^2+^	57.3
Mn^2+^	56.6
Mg^2+^	55.8
Na^+^	54.0
Li^+^	53.7

### Overall Structure of the N-terminal Domain of Sgo0707

**Figure 5:**
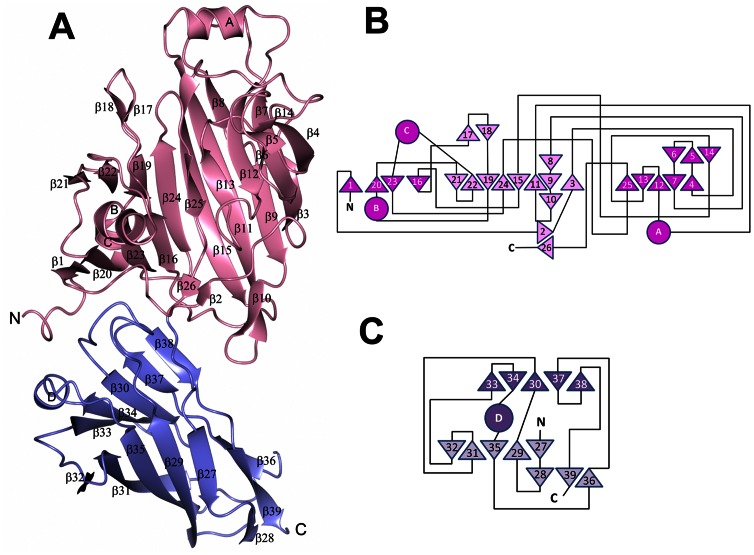
The overall structure of Sgo0707N. A: Ribbon representation of Sgo0707N. The N1-domain is coloured pink and the N2-domain purple. B: Topology diagram of Sgo0707N1. C: Topology diagram of Sgo0707N2. β-strands are depicted as triangles and α-helices as circles. The N1- and N2-domains are shown in pink and purple respectively.

A construct of Sgo0707N representing residues 36–458 was expressed, purified and crystallized. The structure of *S. gordonii* Sgo0707N was solved to a resolution of 2.4 Å by single anomalous dispersion using one crystal of SeMet substituted protein belonging to the *I*222 space group with four molecules in the asymmetric unit. The initial model was subsequently refined against native data to 2.1 Å resolution. The final model has an average B-factor of 29.5 Å^2^, calculated on all protein atoms, and a crystallographic R-factor of 17.9% (R_free_ = 22.5%). The model consists of amino acids 36–455 as well as 6–12 residues from the preceding linker. No, or weak, electron density was observed for the loop residues 427–430. The structure can be divided into two domains, N1 and N2, which both adopt β-sandwich folds with antiparallel β-sheets (Fig. 5). The N1-domain, (residues 36–311), comprises a β-sandwich built up from two sheets consisting of nine (S1) and eight (S2) strands respectively. The first strand of S1, β1, is connected to β3 via a long segment that runs across the domain over to the S2 sheet. The S1 sheet is broken with four strands on one side (S1a) and five strands on the other (S1b). A small subdomain (SDA), mostly consisting of the α-helix (αC) that connects β22 with β23 and the first part of the N-terminal segment that runs across the β-sheet, is located over the S1a sheet. SDA also comprises a loop containing a small β-hairpin β17/β18. On top of S1b another small subdomain (SDB) is located, consisting of a loop segment connecting β4 and β7, a coiled segment connecting β13 and β15 and a helix, αA, connecting β11 and β12. In addition three short β-strands, β5, β6 and β14, form a small β-sheet. The N2-domain (residues 312–456) consists of two β-sheets of five β-strands each. The domain also comprises a small three-stranded β-sheet at the corner of the β-sandwich. The loops connecting the β-strands on the side facing the N1-domain are long and one contains a short α-helix (αD). In three of the four molecules in the asymmetric unit the linker proceeding the cloned construct is visible, six residues in molecule A, and 11–12 residues in molecule B and C. In B and C the linker folds as a β-strand (β0) that becomes the first of the S1 sheet. The extra strand, β0, participates in crystal packing by linking the S1a sheets of two molecules, A with C and B with D.

### A Putative Binding Cleft in the N1-domain

**Figure 6:**
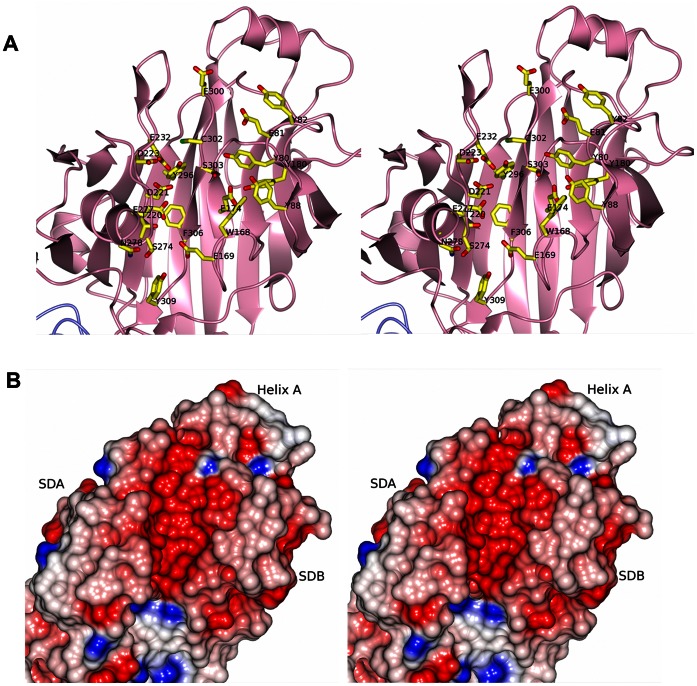
The putative binding cleft in the N1-domain. A: Stereo representation of the cleft in the N1-domain. Amino acids in the pocket are represented as cylinders and are labeled. B: The N1-domain is presented as an electrostatic surface, colored in red and blue according to negative and positive electrostatic potential, respectively. The cleft has considerable negative electrostatic potential. The figure is shown in stereo.

An open cleft is formed between the subdomains SDA and SDB and the split between β16 and β25 in the S1-sheet (Fig. 6a). The underlying S2 sheet constitutes the floor of the cleft. Furthermore, the N-terminal segment that runs across the domain and a small anti-parallel β-sheet consisting of stands β2 and β26 form the rim of the cleft. As calculated by the CASTp server [38] the cleft has a volume of 333 Å^3^.

The pocket exhibits a predominantly negative surface potential (Fig. 6b) formed by Glu81, Glu169, Glu174, Glu232, Asp221, Asp223, Glu277, Glu300 together with the polar residues Thr220, Asn278 Ser274, Ser303 and Cys302. The cleft is also built up from several aromatic residues, Tyr80, Tyr82, Tyr88, Trp168, Tyr180, Tyr296, Phe306, Tyr309, mainly originating from the loops of SDB.

In all four chains positive electron density is found in the cleft but this is more pronounced in chains A and D. Although the protein was crystallized without the addition of metal ions positive electron density, indicating a partly occupied metal ion was observed in association with Asp221 (OD1), Asp223 (OD2) and Thr271 (O) of the A-chain. The electron density in the corresponding sites in chains B–D was less resolved. The putative metal is further coordinated by two water molecules and what was modeled as a sulfate ion. An additional stretch of positive electron density is observed in the binding cleft, stacked between the side chains of Tyr80, Tyr88 and Trp168. In chain A, it is unambiguous that this density originates from the final C-terminal residues 456–458 (YEK) from a symmetry related molecule. The density is however not of sufficient quality for the side chains to be modeled with certainty. Residual electron density is also observed in the cleft of chain C although no symmetry related protein chains are present in the close vicinity.

### Structural Relatives of Sgo0707N

**Figure 7:**
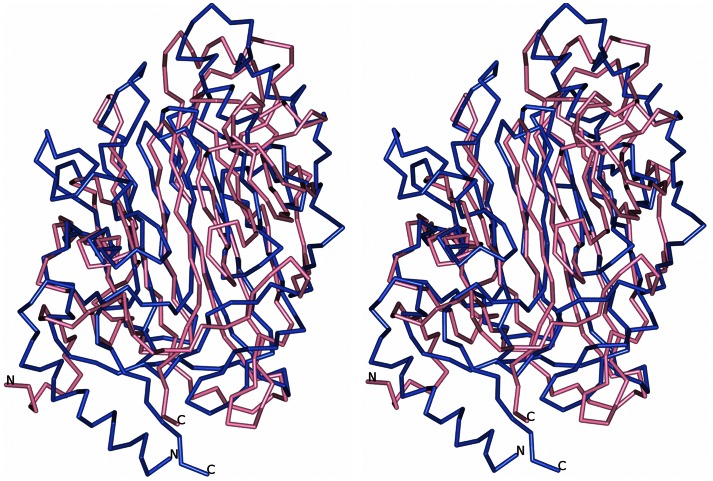
Superposition of Sgo0707N1 and the V-domain of SspB. The N1-domain is superimposed on the variable domain of SspB from *S. gordonii*. Sgo0707 is depicted in pink and SspB in blue. The figure is shown in stereo.

A Dali server search [39] was carried out on the N1- and N2-domains separately. Surprisingly the closest structural relatives of the N1-domain were identified as the variable domains of the AgI/II proteins SspB from *S. gordonii* (PDB code 2WD6 [40]) and SpaP from *Streptococcus mutans* (PDB code 1JMM [11], [41]). Both share approximately 10% sequence identity with Sgo0707N1 with a root mean square deviation (r.m.s.d.) of 3.7 Å and a Z-score of 12.2 and 11.0 respectively. A comparison between the Sgo0707 N1-domain and the variable domain of SspB is shown in Fig. 7.

The structural similarities include the size of the putative binding cleft, 333 and 418 Å^3^ respectively for Sgo0707N1 and SspB, whereas the cleft in SpaP is larger, 2280 Å^3^. However, whereas SspB and SpaP have a tightly bound metal ion in the cleft, Sgo0707N1 does not. In addition, the cleft of Sgo0707N1 has a more predominant negative charge than the two AgI/II proteins.

The N2-domain consists mainly of two β-sheets of five β-strands each forming an IgG-like domain of CnaA fold. The Dali server search of the N2-domain recognized the structure of the middle domain of the *Bacillus cereus* BcpA pilin (PDB code 3KPT [42]) as the closest structural relative with a Z-score of 16.6, 15% sequence identity of 131 aligned C_α_ atoms and an r.m.s.d. of 2.3 Å. In general all hits in the Dali search represent IgG-like modules that build up surface proteins on Gram-positive bacteria.

Interestingly, many structures in this group are stabilized by intra-molecular isopeptide bonds [10] between the side chains of a lysine and an asparagine or an aspartic acid. Such bond formation is facilitated by a close aspartic or glutamic acid and is believed to protect the protein from mechanical and chemical stress. No isopeptide bonds are however observed in the Sgo0707 N2-domain and the residues in the equivalent lysine-asparagine positions are Leu319 and Asn434. The position where a catalytic acid would be expected is occupied by Gln360. Thus the N2-domain lacks the prerequisites for isopeptide bond formation.

### Collagen Docking

**Figure 8:**
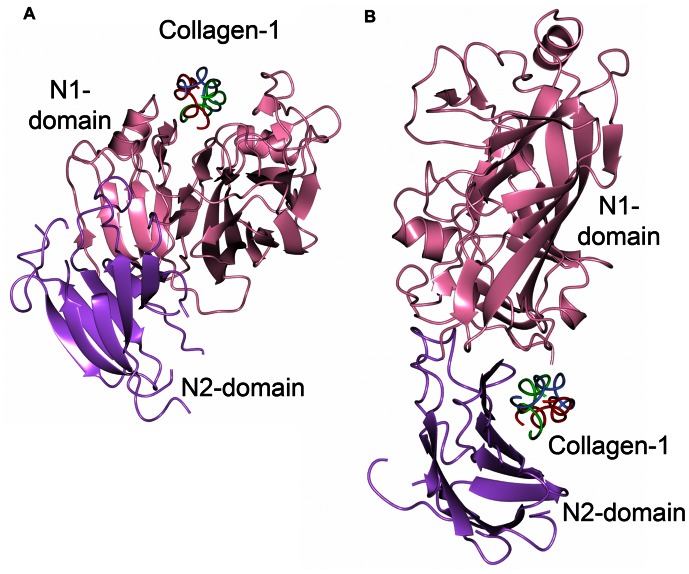
Sgo0707 collagen docking. A model of collagen docked to the Sgo0707 crystal structure using Firedock. A: The collagen triple helix is docked into the binding pocket of the N1-domain. B: Collagen is docked in the groove formed by the interface between the N1- and N2-domains.

Three web-based docking programs (GRAMMx, HexServer and Firedock) were used to dock a type-1 collagen fragment to the Sgo0707N crystal structure. The programs were consistent in predicting two areas of the protein as putative interaction sites. Firstly, the groove in the N1-domain, discussed above as a putative binding cleft, and secondly the concave surface formed by the S4 sheet of the N2-domain and loops from the N1-domain (Fig. 8). By superimposing the collagen binding proteins ACE19 of *Enterococus faecalis* [43] and CNA of *Staphylococcus aureus* [37] onto Sgo0707N it could be shown that the collagen triple helix was docked onto the opposite side of the β-sandwich in Sgo0707N compared to that observed for the collagen binding proteins.

## Discussion

In a previous study, using a ΔSrtA mutant, we identified a novel LPXTG-linked protein, Sgo0707, from the growth medium [16]. The Sgo0707 protein consists of 1643 amino acids including a leader peptide, an N-terminal region, eight 84-amino acid repeat domains, five 88-amino acid repeat domains and a C-terminal domain containing the LPXTG sorting motif. The domain organization is similar to that of the fibrillar protein CshA from *S*. *gordonii* [13]. In a study by McNab and coworkers, CshA was shown to be involved in bacterial binding to fibronectin [44], an interaction which could be blocked using antibodies recognizing the N-terminal domain (amino acids 42–886) of the protein. Based on their related domain organization, our hypothesis was that the N-terminal region of Sgo0707 also represents an adhesive domain. Therefore we chose to investigate the structural and functional characteristics of the N-terminus of Sgo0707.

*S. gordonii* is known to express a number of multi-ligand adhesins, covalently bound to the cell wall. In this study, through selective extraction of cell wall proteins, Sgo0707 was revealed as an abundant component of the cell wall proteome. Therefore we investigated the binding of Sgo0707 to a range of proteins found in the oral cavity. For saliva- or serum-coated surfaces, no significant difference was seen between the wild-type strain and an isogenic ΔSgo0707 mutant. Similarly, no differences in auto-aggregation were seen. Interestingly, binding to type-1 collagen was significantly reduced in the mutant strain. These data suggest that Sgo0707 plays a role in initial adherence of *S. gordonii* to type-1 collagen. Interactions between the wild-type strain and collagen-coated surfaces could be competitively inhibited using a recombinant polypeptide encompassing residues 36–458 of Sgo0707, suggesting that binding is mediated via the N-terminal region. However, binding was not completely abolished even at high concentrations of the recombinant protein confirming that other cell surface proteins of *S. gordonii* also bind collagen. This is in agreement with previous studies showing that SspA and SspB, members of the AgI/II family [45], bind type-1 collagen [46]. Thus, as proposed previously, *S. gordonii* shows redundancy with respect to cell surface adhesins.

The crystal structure of amino acids 36–455 from the N-terminal of Sgo0707 revealed that it was divided into two domains, N1 and N2, that mainly consist of β-strands. The N1-domain is the largest domain (residues 36–311) harbouring a putative binding cleft which strengthens the original hypothesis of the N-terminal, non-repetitive domain, functioning as an adhesin. To our surprise we found that the N1-domain, despite very low sequence similarity, exhibits a striking structural similarity to the variable domains of the AgI/II adhesins, also expressed by oral streptococci. Despite having fundamentally different domain organizations; the AgI/II variable domain is located in the centre of the protein but presented at the tip due to intertwining flanking regions and the formation of a fibrillar structure [11], and Sgo0707N1 being expressed as the N-terminal domain followed by a repetition of C-terminal domains, they both obtain similar folds, including putative binding clefts. This points to an interesting divergent evolution, possibly due to gene duplication of a domain hitherto only found in oral streptococci. The AgI/II adhesins are the best studied adhesins expressed by oral bacteria and several interaction partners are known, such as gp340, type-1 collagen, integrin and the Mfa1 fimbria from *Porphyromonas gingivalis* [47], however a ligand specific for the variable domain has never been described. Despite some similarities the binding pockets of the AgI/II proteins (SspB and SpaP) and Sgo0707 also show significant differences. Whereas the Sgo0707 cleft is predominantly negatively charged the AgI/II clefts are less charged and have a tightly bound metal ion. Thermal melting analysis showed that Sgo0707 is indeed stabilized by Ca^2+^-ions, as is SspB [40] and electron density also indicates the presence of metal ions also in the Sgo0707 cleft. However, the electron density is not of such quality that it can be concluded that a true metal binding site is present. The AgI/II variable domains have been proposed to be carbohydrate binding [41] but in a previous study we performed a glycan array screen for the variable domain of the AgI/II adhesin SspB which showed that the domain most likely does not interact with carbohydrates [40]. A similar screen with Sgo0707N gave the same negative result. However, these screens do not rule out that the adhesins may interact with carbohydrates not included in the array or with glycoproteins. In addition, the two forms of adhesins are related with regards to collagen binding. AgI/II shows affinity for type-1 collagen, but the interaction is not described in molecular detail. Similarly, Sgo0707 interacts with type-1 collagen but the interaction surface is not obvious. A docking of collagen to Sgo0707N yielded two different suggestions. The first alternative presented the collagen triple helix bound along the putative binding cleft in the N1-domain and as a second alternative collagen was placed at the concave side of the S4-sheet of the N2-domain, at the interface between the N1- and N2-domains. By looking at the electrostatic surfaces of the two sites it is obvious that the characteristics of the two suggested sites are not consistent. Whereas the cleft in the N1-domain is predominantly negatively charged, the concave side of the S4-sheet in the N2-domain is mostly non-polar and shows more resemblance to the binding surfaces of the collagen-binding proteins CNA and ACE. However in those proteins, the collagen triple-helix is bound on the opposite side of the β-sandwich (corresponding to the S3 sheet of Sgo0707) and is embraced by a linker that connects two domains. In Sgo0707, collagen is modelled to bind to the S4 sheet and interacts with loops and turns from the N1-domain. This putative interaction cannot be as tight as the “collagen hug” model in the true collagen-binding proteins. Another mechanism for collagen adhesion, observed both in eukaryotes [48] and the *Streptococcus pneumoniae* pilus adhesin RrgA [49], involves the interaction between collagen and a MIDAS motif coordinated metal ion. However, neither a high occupancy metal ion, nor a MIDAS motif is found in Sgo0707.

Binding to collagen is generally not an interaction that is necessary for normal colonization of the oral cavity, however it may be an important invasion mechanism at non-oral sites. The oral streptococcal adhesin most studied with regards to pathogenicity is GspB. Its binding to the platelet membrane glycoprotein GPIbα is implied to be an important factor for causing infective endocarditis [6]. Similarly the collagen binding properties of oral streptococci are considered important virulence factors for infective endocarditis [50]. Interestingly the collagen-binding protein Cnm, expressed by certain serotypes of the major pathogen of dental caries, *S. mutans*, was recently identified as an important factor in the onset of hemorrhagic stroke [51]. Although collagen may not be the main ligand for Sgo0707, its ability to interact (non-specifically) with exposed collagen tissue may be of importance when attaching to non-oral surfaces.

We also show that Sgo0707 makes significant contributions in binding to oral keratinocytes, the deletion mutant showed a 30% decrease in binding compared to wild-type. The particular molecule that is recognized on the keratinocyte cell surface cannot be determined at this point, however these cells are known to express a wide variety of surface molecules, for example integrins, cadherins, selectins, and members from the immunoglobulin superfamily [52].

Interestingly, the 1643 amino acid long protein contains only one cysteine, Cys302, and that single residue is located in the floor of the putative binding cleft in the N1-domain. In the *S. pyogenes* pilus adhesin Cpa [53] there is also a single cysteine in a putative binding cleft. In the crystal structure of Cpa, the cysteine forms an intra thioester bond with a glutamine in the same cleft, priming for a reaction with a host cell ligand and a subsequent covalent linkage. The authors show that the involved residues indeed are important for the attachment to host cells. The position of the cysteine in Sgo0707N1 similarly implies a crucial function where the residue may be involved in interacting with host cell ligands or to surface molecules of other bacteria in the oral biofilm. However, no internal thioester is formed in Sgo0707N1 and further studies are needed to examine if the cysteine has a role in ligand recognition.

In conclusion, the oral biofilm is known to harbor hundreds of different bacterial strains and oral streptococci, such as *S. gordonii,* are among the most abundant. Due to the complex environment oral streptococci can be expected to have multiple co-aggregation partners and hosts. The possibility of Sgo0707 having a palette of ligands and multiple binding activities is therefore to be expected. We suggest that Sgo0707 may have a specific set of binding partners that is recognized via the binding pocket in the N1-domain but that it will be a considerable challenge to identify these ligands. We also suggest that the protein interacts non-specifically with other ligands such as the interaction with type-1 collagen that is described here. The ability to bind to collagen may however be very important for the bacteria when located at non-oral sites such as the heart valves. In addition the Sgo0707N crystal structure that is presented here constitutes only a fraction of the total protein. The C-terminal repeats that are believed to build up the stalk of the protein are not yet characterized but may be equally important for the function of the protein.
